# White dot in the eye

**DOI:** 10.4103/0974-620X.48424

**Published:** 2009

**Authors:** David S. I. Taylor

**Affiliations:** International Council of Ophthalmology, UK

An 8-week-old infant was brought to the clinic by her parents, who had recently noticed a white dot in the right eye of their child.

## Questions

1. What is the diagnosis? What are the radiating lines?
2. What is the management?

## Answers

Diagnosis:Congenital Anterior Polar Cataract with Ghost Vessels (remnants of the pupillary membrane)

Management:Cycloplegic refraction, patching of the normal left eye, periodic (every 3 months) review, to follow visual development, and note progression, if any.

## Comments

**Figure 1:**
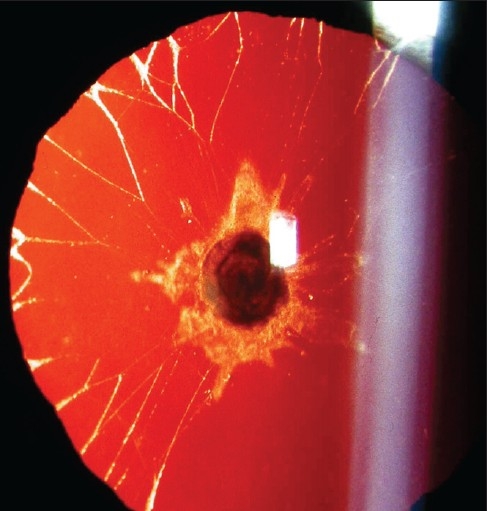
Slit lamp photograph – retroillumination. The pupil has been dilated

Lens opacities in infancy have a wide spectrum of presentations. Anterior polar cataracts usually appear as a tiny white dot on the anterior surface of the lens in the axial area; but vary in size from one case to another. Sometimes anterior polar lens opacities assume a plaque-like appearance [Figure 1]; these are often associated with remnants of the pupillary membrane. Their embryological origin is believed to be different from the dot like anterior polar cataracts: the dot-like opacities are related to abnormalities of lens vesicle detachment, whereas the plaque-like ones are caused by abnormalities of pupillary membrane regression. Some cases are inherited as an autosomal dominant trait. They have also been reported in association with Peters’ anomaly and aniridia.

Often the cataracts are not visually significant; however, they may be associated with refractive errors that can cause amblyopia and strabismus. Cycloplegic refraction is therefore essential. Associated astigmatism may be secondary to radial capsular wrinkling. Occlusion therapy may be required in unilateral cases to prevent amblyopia. All cases should be followed throughout their years of visual development. Anterior polar cataracts are usually static. If they progress and become visually significant, dilating the pupils may sometime improve vision. Surgical intervention may be necessary if conservative measures to facilitate visual development fail.

